# Plasma Dimethylarginine Levels and Carotid Intima–Media Thickness are related to Atrial Fibrillation in Patients with Embolic Stroke

**DOI:** 10.3390/ijms20030730

**Published:** 2019-02-09

**Authors:** Gerrit M. Grosse, Saskia Biber, Jan-Thorben Sieweke, Jens Martens-Lobenhoffer, Maria M. Gabriel, Anne-Sophie Putzer, Isabel Hasse, Till van Gemmeren, Ramona Schuppner, Hans Worthmann, Ralf Lichtinghagen, Stefanie M. Bode-Böger, Udo Bavendiek, Karin Weissenborn

**Affiliations:** 1 Department of Neurology, Hannover Medical School, 30625 Hannover, Germany; saskia.biber@stud.mh-hannover.de (S.B.); gabriel.maria@mh-hannover.de (M.M.G.); anne-sophie.putzer@stud.mh-hannover.de (A.-S.P.); hasse.isabel@mh-hannover.de (I.H.); till.v.gemmeren@stud.mh-hannover.de (T.v.G.); schuppner.ramona@mh-hannover.de (R.S.); worthmann.hans@mh-hannover.de (H.W.); weissenborn.karin@mh-hannover.de (K.W.); 2 Department of Cardiology, Hannover Medical School, 30625 Hannover, Germany; sieweke.jan-thorben@mh-hannover.de (J.-T.S.); bavendiek.udo@mh-hannover.de (U.B.); 3 Institute of Clinical Pharmacology, Otto-von-Guericke University Magdeburg, 39120 Magdeburg, Germany; jens.martens-lobenhoffer@med.ovgu.de (J.M.-L.); stefanie.bode-boeger@med.ovgu.de (S.M.B.-B.); 4 Institute of Clinical Chemistry, Hannover Medical School, 30625 Hannover, Germany; lichtinghagen.ralf@mh-hannover.de

**Keywords:** arginine, atrial fibrillation, dimethylarginine, embolic stroke, endothelial dysfunction, ESUS, intima–media thickness

## Abstract

A relevant part of embolic strokes of undetermined source (ESUS) is assumed to be due to non-detected atrial fibrillation (AF). In this study, we aimed to investigate if markers of endothelial dysfunction and damage may indicate AF risk in embolic stroke. Eighty-eight patients with ischemic stroke confirmed by imaging were assigned to one of three groups: ESUS, AF, or micro-/macroangiopathy. ESUS patients underwent prolonged Holter electrocardiography scheduled for three days. The National Institutes of Health Stroke Scale (NIHSS), the CHA_2_DS_2_VASC score, and the carotid intima–media thickness (CIMT) were obtained. Markers of endothelial (dys)function (L-arginine, asymmetric dimethylarginine (ADMA), symmetric dimethylarginine (SDMA)) were measured at day seven after stroke. ESUS patients were younger and had fewer cardiovascular risk factors than patients with determined stroke etiology. Compared with AF patients, ESUS patients showed significantly lower values of SDMA (*p* = 0.004) and higher values of L-arginine (*p* = 0.031), L-arginine/ADMA ratio (*p* = 0.006), L-arginine/SDMA ratio (*p* = 0.002), and ADMA/SDMA ratio (*p* = 0.013). Concordant differences could be observed comparing ESUS patients with those with newly diagnosed AF (*p* = 0.026; *p* = 0.03; *p* = 0.009; *p* = 0.004; and *p* = 0.046, respectively). CIMT was significantly larger in AF than in ESUS patients (*p* < 0.001), and was identified as an AF risk factor independent from CHA_2_DS_2_VASC in the regression analysis (*p* = 0.014). These findings may support future stratification for AF risk in patients who have suffered embolic stroke.

## 1. Introduction

About 25% of all ischemic strokes remain unexplained despite intensive diagnostic workup. In these cryptogenic strokes, there is uncertainty regarding drug regimen for secondary stroke prevention due to lack of knowledge concerning the underlying pathophysiology. In 2014, Hart et al. proclaimed a new entity of cryptogenic strokes with an embolic stroke pattern in magnetic resonance (MRI) or computed tomography (CT) imaging: the so-called “embolic stroke of undetermined source” (ESUS) [1]. It is presumed that a relevant part of ESUS is due to non-detected paroxysmal atrial fibrillation (AF). Thus, multicenter clinical trials have been initiated to investigate the potential advantage of non-vitamin K oral anticoagulants (NOAC) over acetylsalicylic acid (ASA) in ESUS. However, in the NAVIGATE-ESUS study, no benefit of Rivaroxaban could be found compared with ASA for secondary stroke prevention in ESUS patients [2]. This result indicates that proper diagnostic workup to identify paroxysmal AF in ESUS may still be requested as a precondition for the initiation of anticoagulant therapy.

In clinical practice, long-term (Holter) electrocardiography (ECG) or implantable loop recorders are used for the detection of paroxysmal AF. Wachter et al. recently showed that proper diagnosis of AF is highly dependent on the recording time of long-term ECG [3]. Alternative implantable loop recorders require an invasive procedure and confer a potential risk of infection. Thus, current diagnostic procedures are accompanied by disadvantages in practicability. More reasonable biomarkers supporting the diagnosis of AF in ESUS patients would therefore be highly advantageous but are currently not available.

There is growing evidence that AF is a systemic disorder which is not limited to the left atrium but driven by inflammatory and endothelial disorder mechanisms [4,5,6]. However, it remains unclear whether endothelial damage leads to AF or vice versa. There are hints that atrial fibrosis which develops as a consequence of higher arterial stiffness and consecutive higher cardiac afterload [7,8] plays a role in the pathophysiology of AF. On the other hand, markers of endothelial dysfunction normalize in AF patients after successful cardioversion or ablation [9,10]. In contrast, in a study by Tveit et al., L-arginine and asymmetric dimethylarginine (ADMA) were unaffected after cardioversion in AF patients and did not differ on follow-up between patients with persistent sinus rhythm and those with relapse of AF [11]. Thus, current findings are contradictory.

Dimethylarginines are known to be closely associated with endothelial dysfunction. ADMA is an inhibitor of nitric oxide (NO) synthesis as it competes with its analogue L-arginine at the NO-synthase. Several studies have described an association of ADMA with cardiovascular risk [12], carotid intima–media thickness (CIMT) [13], and pathologies like ischemic [14,15] and hemorrhagic stroke [16]. Symmetric dimethylarginine (SDMA), which is an isomer of ADMA, modulates the synthesis of NO via inhibition of cellular uptake of L-arginine. SDMA levels have been previously found to be closely associated with renal function [17,18]. While there are fewer studies investigating the role of SDMA in cardiovascular risk compared with ADMA [12,19], there is evidence of SDMA being a relevant marker of cardiovascular diseases, independently from renal function [17]. Schulze et al. showed that patients with AF-related ischemic strokes present significantly lower L-arginine/SDMA ratios than patients with other stroke etiologies [20].

In conclusion, dimethylarginines are of high interest in investigating ESUS and etiologies of ischemic stroke. In this study, we therefore investigated L-arginine, ADMA, SDMA, and CIMT as markers of endothelial (dys)function and subclinical atherosclerosis in patients with ESUS and those with determined stroke etiology, i.e., AF as well as angiopathy. We hypothesized that stroke patients with underlying AF or angiopathy would display higher values of endothelial dysfunction parameters than patients with ESUS, aiming to find novel markers to support the diagnosis of stroke etiology and, moreover, to gain further insight into the underlying pathophysiology of ESUS.

## 2. Results

### 2.1. Epidemiological Data

For our epidemiological data, see Table 1. In total, 88 patients were considered. Fifty-nine patients were initially admitted with ESUS, of whom 16 showed AF in ECG on admission or in long-term ECG as the most likely etiology (newly diagnosed AF), leaving 43 cases in which no etiology could be determined. Nine patients suffered from previously diagnosed AF and 20 patients had stroke due to angiopathy. Significant differences between the study groups were observed for age, sex, the Essen Stroke Risk Score (ESRS), CHA_2_DS_2_VASC, and the National Institutes of Health Stroke Scale (NIHSS). Patients with ESUS showed the most favorable risk profile as determined by CHA_2_DS_2_VASC and ESRS. Other baseline characteristics did not significantly differ. In 85 cases, stroke diagnosis was confirmed with diffusion-weighted (DWI) magnetic resonance imaging (MRI), and in 3 cases with previously known AF using CT and CT angiography. The median duration of the Holter ECG was 72 h for ESUS patients.

### 2.2. Plasma Dimethylarginine Levels in Ischemic Stroke of Different Causes

Significant group differences were detected using the Kruskal–Wallis test for SDMA levels (*p* = 0.018), L-arginine/ADMA ratio (*p* = 0.045), and L-arginine/SDMA ratio (*p* = 0.016). Intergroup differences were found in comparison of ESUS with AF in total and with newly diagnosed AF regarding L-arginine, SDMA, L-arginine/ADMA ratio, L-arginine/SDMA ratio, and ADMA/SDMA ratio (see Table 2).

Comparison of ESUS with angiopathic strokes showed significant differences exclusively concerning SDMA (*p* = 0.037), with SDMA levels being higher in angiopathic strokes. There were no significant differences between AF in total and angiopathic strokes regarding L-arginine or dimethylarginines.

### 2.3. L-arginine/SDMA Ratio as A Potential Marker for Identifying AF in Patients Admitted with ESUS

**Figure 1:**
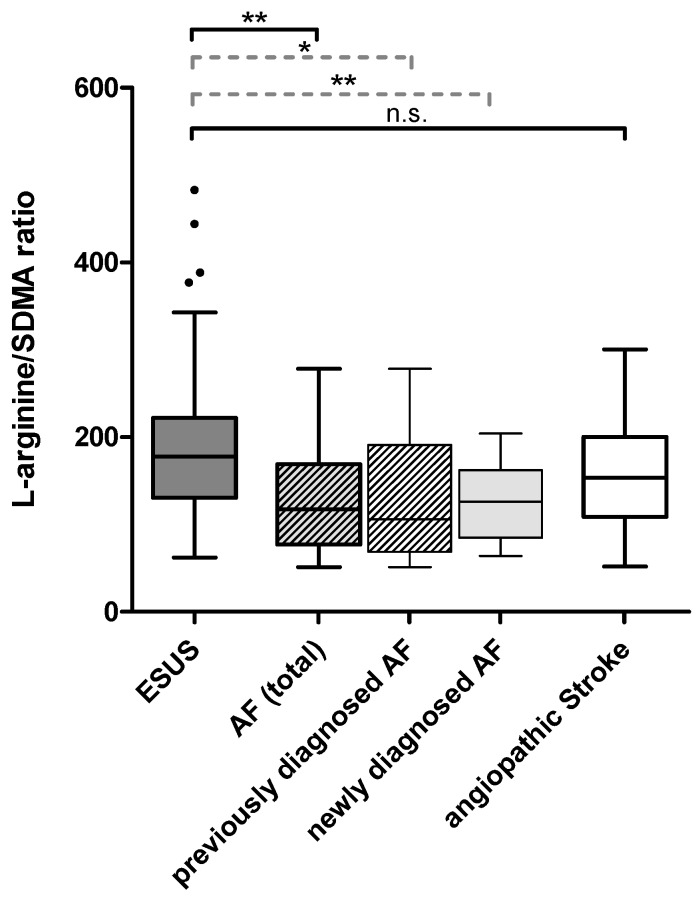
Group differences of L-arginine/SDMA ratio. AF (total) subsumes previously and newly diagnosed AF. * *p* ≤ 0.05 ** *p* ≤ 0.01; n.s.: not significant.

**Figure 2:**
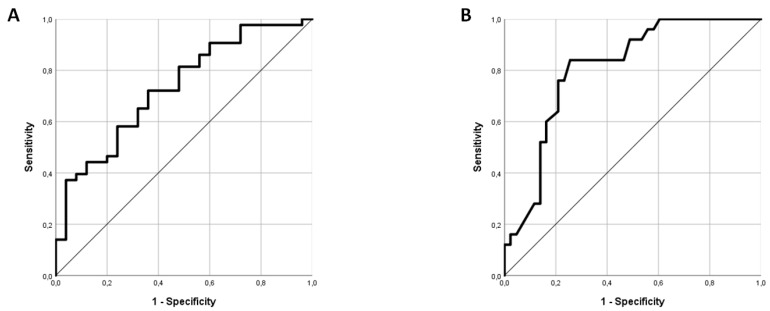
ROC analysis of L-arginine/SDMA ratio (A) and CIMT (B) as distinguishing markers between AF and ESUS patients. a: AUC: 0.732; (95% CI: 0.610–0.854; *p* = 0.002); b: AUC: 0.807 (95% CI: 0.704–0.910; *p* < 0.001).

As the L-arginine/SDMA ratio turned out to be particularly different between ESUS and AF patients (Figure 1), this parameter was further evaluated using backwards binary logistic regression. In an analysis including L-arginine/SDMA ratio, CHA_2_DS_2_VASC (as a composite score including age, sex, and thrombembolic risk factors), NIHSS, and serum creatinine, L-arginine/SDMA ratio was found to not be independently associated with newly diagnosed AF with a remaining difference by tendency (*p* = 0.099), whereas CHA_2_DS_2_VASC proved significantly different (*p* = 0.02). Receiver operating characteristic (ROC) analysis revealed moderate discriminability of AF and ESUS through the L-arginine/SDMA ratio with an area under the curve (AUC) of 0.732 (95% CI: 0.610–0.854) (Figure 2A).

In patients who underwent transesophageal echocardiography (TEE) there was no significant difference between patients with (*N* = 15) or without diagnosis of patent foramen ovale (PFO) (*N* = 29) regarding dimethylarginines or CIMT (data not shown). Also, in the subgroup analysis of ESUS patients, there were no significant differences between these groups (*N* = 10 vs. *N* = 21).

### 2.4. Carotid Intima–Media Thickness Differs between Patients with ESUS and Other Etiologies

**Figure 3:**
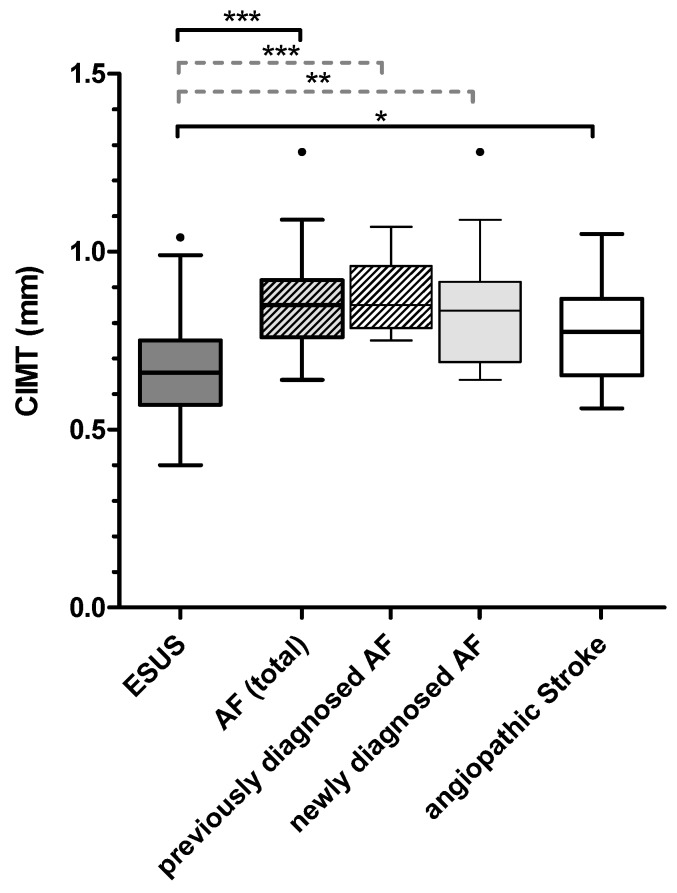
Group differences in CIMT. AF (total) subsumes previously and newly diagnosed AF. * *p* ≤ 0.05; ** p ≤ 0.01; *** p ≤ 0.001.

For group comparison of CIMT, see Table 2. Patients with known, newly diagnosed AF and all patients with AF showed significantly higher values of CIMT than patients with ESUS (*p* < 0.001, *p* = 0.001, and *p* < 0.001, respectively). Moreover, patients with angiopathic strokes had significantly thicker CIMT than ESUS patients (*p* = 0.021) (Figure 3).

Including CHA_2_DS_2_VASC, binary logistic regression revealed an independent association of CIMT with AF in the whole cohort (*p* = 0.014) and a non-significant trend considering newly diagnosed AF patients (*p* = 0.06). ROC analysis showed a good discriminability of AF and ESUS through CIMT with an AUC of 0.807 (95% CI: 0.704–0.910) (Figure 2B). At a CIMT cutoff of 0.7 mm, the sensitivity was 84% and the specificity was 60.5%; at a cutoff of 0.8 mm, the sensitivity was 60% and the specificity was 83.7%.

### 2.5. Markers of Endothelial Dysfunction and Thrombembolic Risk

**Figure 4:**
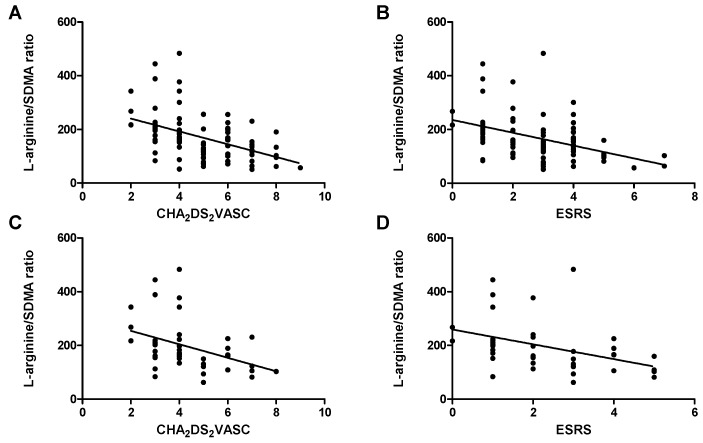
Correlation of L-arginine/SDMA ratio with CHA_2_DS_2_VASC (**A**) and ESRS (**B**) in the whole sample and within the ESUS collective (**C**,**D**). A: Spearman’s r: −0.50, *p* < 0.001; B: Spearman’s r: −0.46, *p* < 0.001; C: Spearman’s r: −0.47, *p* = 0.002; D: Spearman’s r: −0.53, *p* < 0.001.

Within the whole study cohort, associations between dimethylarginines, CIMT, and cardiovascular risk scores could be identified (see Table S1 in the supplemental material). After Bonferroni correction there was a significant positive correlation of both dimethylarginines and CIMT with CHA_2_DS_2_VASC and ESRS, as well as an inverse association of these scores with L-arginine/SDMA ratio (Figure 4A,B), L-arginine/ADMA ratio, and ADMA/SDMA ratio. These associations were mostly present within the cohort of patients who were discharged with ESUS diagnosis (see Table S2 and Figure 4C,D). 

### 2.6. Markers of Endothelial Dysfunction and Renal Function

After Bonferroni correction, there was a significant positive correlation between serum creatinine levels and SDMA, and inverse correlations of serum creatinine with L-arginine/SDMA ratio, L-arginine/ADMA ratio, and ADMA/SDMA ratio. Same associations were observed for the comparison with the estimated glomerular filtration rate (eGFR). In addition, eGFR was positively correlated with L-arginine (see Table S3).

## 3. Discussion

In this study, we investigated markers of endothelial dysfunction in patients with ESUS, cardioembolic stroke due to AF, and stroke due to angiopathy. A proportion of 27.11% of the admitted ESUS patients were newly diagnosed with AF. This is comparable to the results of a recent study by Israel et al. who provided ESUS patients with implantable loop recorders. In this setting, in 23.6% of ESUS patients, AF was detectable [21]. It needs to be taken into account that in our current study, patients with AF diagnosed through ECG on admission were included in the newly diagnosed AF group, which might lead to a higher AF identification rate than that in the study by Israel et al. We found that ESUS patients were remarkably younger and had significantly fewer cardiovascular risk factors than patients with determined stroke etiology, which is in accordance with several studies investigating characteristics of ESUS patients [21,22,23]. Interestingly, Martinez-Majander et al. described a comparable risk of stroke recurrence between ESUS patients and those who suffered stroke due to cardioembolism [22] and concluded that this might hint at a similar pathophysiology of the two entities. Similar results have been reported by Arauz et al. [24]. Of note, both studies report that ESUS patients suffer from strokes of lower severity—a finding that is in line with our work. Another large cohort study also reported highly different amounts of cardiovascular risk factors between patients without, with known, and with newly diagnosed AF [25]. Upon analysis of the same cohort it was recently stated that, in accordance with our results, the CHA_2_DS_2_VASC score might be useful for predicting AF in ischemic stroke patients [26].

One of the main findings of our current study is that patients with ESUS show significantly different L-arginine and dimethylarginine levels than patients who suffered stroke due to AF. L-arginine was significantly higher, whereas SDMA levels were significantly lower in ESUS patients. Ratios of L-arginine to dimethylarginines were concordantly different, with marked differences concerning L-arginine/SDMA ratio. However, no differences could be observed for ADMA alone. In ESUS patients, the values of L-arginine/SDMA ratio were within the previously reported 2.5%–97.5% percentiles of normal values [27], while those of AF patients in total and newly diagnosed AF patients were not. Importantly, our findings are clearly in line with those of Schulze et al. who described similar differences between stroke patients with AF and those with sinus rhythm [20]. However, no further subgroup analysis regarding cryptogenic strokes has been reported by Schulze et al. [20].

As shown previously, dimethylarginines are closely related to cardiovascular risk. Also, in our cohort these markers strongly correlated with both thrombembolic risk scores CHA_2_DS_2_VASC and ESRS. Therefore, it cannot be ruled out that the group differences described might be due to these associations. However, if the group differences are solely based on cardiovascular risk, it should have been expected that there would be differences as well concerning ADMA, since this marker was redundantly reported to be closely associated with cardiovascular risk factors. However, in our current study no group differences could be observed between the study groups regarding ADMA at all, despite the divergent amount of risk factors. We performed a multivariate logistic regression analysis including CHA_2_DS_2_VASC, serum creatinine, and NIHSS to analyze whether the L-arginine/SDMA ratio was independently different between ESUS and AF patients. However, thrombembolic risk, as measured using CHA_2_DS_2_VASC, turned out to be highly and independently different between the patient groups, as stated above. Thus, we failed to demonstrate a clear independent difference for L-arginine/SDMA ratio as the regression analysis revealed only a non-significant trend for this parameter. ROC analysis showed a moderate discriminability between ESUS and AF using the L-arginine/SDMA ratio. In a large study by Schnabel et al. the authors describe an association of dimethylarginines with AF that was not independent from cardiovascular risk [28]. Of note, it needs to be considered that our results depend on a small but well-selected stroke collective, whereas the analyses by Schnabel et al. were done in the large community-based Framingham study. At the least, the reported trend in our multivariate analysis regarding the difference in the L-arginine/SDMA ratios between ESUS and AF leads to the notion that further studies should be done to prove whether there is an association of the L-arginine/dimethylarginine system with AF in embolic stroke that is independent from cardiovascular risk. Moreover, there are recent reports which may support a possible pathogenetic link of the markers investigated in our study with AF: e.g., SDMA but not ADMA has been described to be associated with left atrial diameter and P wave duration in ECG [29]. Successful occlusion of the left atrial appendage, moreover, led to a significant reduction of SDMA [30], further supporting a pathogenetic link of this distinct marker with atrial cardiopathy. Alterations of the gene coding for Alanine–glyoxylate aminotransferase 2, which is the only enzyme that is involved in metabolizing both ADMA and SDMA, were recently considered to be associated with AF [31].

Of note, the correlation of L-arginine/SDMA ratio with the CHA_2_DS_2_VASC and ESRS was evident within the group of patients who were discharged from hospital with diagnosis of ESUS, implying that follow-up studies might be useful for evaluating this ratio as a predicting marker of recurrent events in ESUS, especially since little is known on how to estimate the individual risk of stroke recurrence in this collective.

Impaired renal function, moreover, is well known to be associated with occurrence of AF [32]. SDMA is considered a reliable marker of renal function [18], being more sensitive than creatinine, and might display also mildly impaired glomerular clearance. Thus, hypothetically, impaired renal function might lead to the accumulation of SDMA [33] that itself contributes to endothelial dysfunction, arterial stiffness, and, finally, to higher risk of AF due to atrial remodeling. Indeed, in the current study, creatinine levels and eGFR were highly positively correlated with SDMA and inversely correlated with L-arginine/SDMA ratio, L-arginine/ADMA ratio, and ADMA/SDMA ratio.

Analogous to the biochemical markers of endothelial dysfunction, CIMT values were differentially distributed among the study collective. ESUS patients showed significantly lower CIMT values than patients with AF in total, patients with newly diagnosed AF, and patients with angiopathic stroke. In the ROC analysis, CIMT showed good results for distinguishing AF from ESUS patients. Moreover, the logistic regression analysis revealed that the difference between ESUS and AF patients in total was independent from thrombembolic risk and age as determined with CHA_2_DS_2_VASC such that our results suggest a role of CIMT in AF beyond this known association. CIMT cutoffs at 0.7 mm and 0.8 mm, respectively, displayed sensitivity and specificity values which may be of interest regarding indication for further rhythm analyses on the search for AF in ESUS patients. Our findings are in accordance with results from a meta-analysis that revealed that higher CIMT values, as well as the presence of carotid plaque and higher arterial stiffness, are associated with enhanced risk for AF [34]. Two other large studies demonstrated an independent relation of CIMT with AF in the general population [35,36]. The findings of our study therefore further support the concept of the closely related pathologies of AF and (subclinical) atherosclerosis/arterial stiffness [37,38,39].

Diverse current publications point to the notion that at least a proportion of ESUS patients display similarities with patients with cardioembolism, e.g., in regard to risk for stroke recurrence or thrombus histology [22,40,41]. Hypothetically, a part of ESUS may be due to embolism based on atrial cardiopathy even beyond AF, which may basically be interpreted as a symptom at the end stage of this pathology [42,43]. Thus, further research to elucidate the mechanisms behind ESUS and its linkage to atrial cardiopathy is highly warranted. In particular, the potential role of endothelial dysfunction markers as indicators of atrial cardiopathy in patients with cryptogenic stroke is yet unclear.

Our study has several limitations. It needs to be taken into account that a possible underlying AF can still not be ruled out in the ESUS group since Holter ECG lasted 72 h in median. In patients with previously known AF, no data on the distinct time point of this diagnosis were available. Longer monitoring, or even implantable loop recorders, would be beneficial in terms of biomarker validation in future studies. The study collective is well defined but relatively small. Thus, the results need to be interpreted with caution and certainly need to be proven in larger studies. Due to the nature of the study we can report only associations and no causality. Possible confounders, especially cardiovascular risk factors and renal function, are discussed above. Finally, though an interfering impact of post-ischemic inflammation on L-arginine and dimethylarginines seems to be unlikely on day 7 after stroke [44], especially since stroke severity in this collective was predominantly mild, we are not able to rule out such a confounding factor.

In conclusion, this is the first study to investigate dimethylarginines and CIMT in patients who suffered ESUS in comparison to those with determined etiology. Patients with known or newly diagnosed AF display higher values of clinical and biochemical markers of endothelial dysfunction and subclinical atherosclerosis than ESUS patients. This may support future stratification for AF risk in patients who have suffered embolic stroke.

## 4. Materials and Methods

### 4.1. Study Population

Seventy-seven patients with acute ischemic stroke who were admitted to the Department of Neurology at Hannover Medical School between August 2016 and April 2017 were prospectively included in the study. Eleven patients with diagnosed AF who were recruited between June 2014 and March 2016 in the course of a former biomarker study by our group investigating acute ischemic stroke were considered in addition. Inclusion criteria were acute focal neurological symptoms with accompanying evidence of cerebral infarction in DWI-MRI or in CT with CT angiography. Exclusion criteria were competing or rare stroke etiologies like vasculitis, endocarditis, dissection of the brain supplying arteries, or evidence of deep vein thrombosis in combination with PFO. The study was approved by the ethics committee at Hannover Medical School (No. 3316-2016). All patients provided written informed consent before inclusion in the study.

### 4.2. Clinical Evaluation

Demographical and clinical data including cardiovascular risk factors were obtained for the whole sample. All patients underwent standard stroke diagnostics including brain imaging (MRI or CT), MR or CT angiography, duplex and Doppler ultrasound of the brain-supplying arteries including measurement of CIMT, and echocardiography. Eighty-eight patients underwent transthoracic echocardiography, and 44 patients of these were diagnosed using transesophageal echocardiography in addition. In the absence of known AF and the absence of AF in standard ECG on admission, patients underwent additional Holter ECG for intended 72 h. Two patients were alternatively diagnosed using a readout of pacemaker recordings.

Patients were assigned to one of the stroke etiologies: microangiopathic stroke, macroangiopathic stroke, cardioembolic stroke due to AF, or ESUS. These were defined as follows: Microangiopathic stroke: DWI-positive lesion in the subcortical space with a maximum diameter of <2 cm without competing stroke etiologies like AF or stenosis of an ipsilateral brain-supplying artery. Macroangiopathic stroke: embolic stroke pattern in DWI (i.e., cortical DWI lesion or subcortical DWI lesion of ≥2 cm) with a stenosis of an ipsilateral brain-supplying artery of ≥50%. Cardioembolic stroke due to AF: embolic stroke pattern in DWI (i.e., simultaneous stroke in different vascular territories, cortical lesion or subcortical lesion of ≥2 cm), or in CT with CT angiography, with evidence of AF in patient’s records (previously known), newly diagnosed in ECG on admission or in Holter ECG. ESUS: embolic stroke pattern in DWI (i.e., simultaneous stroke in different vascular territories, cortical DWI lesion or subcortical DWI lesion of ≥2 cm) without evidence of AF either in ECG on admission or in Holter ECG and without hints of any other specific stroke cause like, e.g., stenosis, vasculitis, or dissection. Assignment to each group was done blinded to the biomarker results. Due to the low number of patients in the macroangiopathic stroke group, micro- and macroangiopathic stroke patients were combined as angiopathic stroke-patients for further analyses.

Thrombembolic risk was evaluated using the CHA_2_DS_2_VASC and ESRS. Stroke severity was determined using the NIHSS on admission.

CIMT was sonographically measured in both common carotid arteries and averaged to a mean value for further analyses.

Serum creatinine levels and eGFR, as calculated using the CKD-EPI equation, were available for all patients.

### 4.3. Biomarker Analysis

Peripheral venous blood was drawn from all patients at 7 days after stroke onset to minimize stroke-related alterations of biomarkers as, e.g., triggered by the ischemic injury. Blood was centrifuged at 1.600× *g* for 15 min. EDTA plasma was stored at −80 °C until analysis. Levels of L-arginine, SDMA, and ADMA were determined in EDTA plasma at the Department of Clinical Pharmacology at the University of Magdeburg using high-performance liquid chromatography–tandem mass spectrometry (HPLC-MS-MS) [45]. In accordance with previous works, ratios were calculated for L-arginine/ADMA, L-arginine/SDMA, and ADMA/SDMA.

### 4.4. Statistical Analysis

Statistical analysis was performed using IBM SPSS Statistics 24 (SPSS Inc., Chicago, IL., USA). Values were tested for Gaussian distribution using the Kolmogorov–Smirnov test. Bilateral comparisons between groups were performed using Student’s *t*-test for normally distributed data or Mann–Whitney *U*-test for non-normally distributed data and Chi-square test for categorical data, as appropriate. Multiple group comparisons were performed using ANOVA or Kruskal–Wallis test. Correlations were calculated with bivariate Pearson correlation or Spearman correlation, as appropriate. For multiple correlation analyses, Bonferroni correction was applied. Binary logistic regression was performed using the stepwise backwards method. ROC analysis was conducted to determine the biomarkers’ ability to discriminate AF from ESUS. A *p*-value of <0.05 was regarded as significant. Figures were created using GraphPad Prism 5 (GraphPad Inc., La Jolla, CA, USA) and IBM SPSS Statistics 24 (SPSS Inc., Chicago, IL., USA). Figures show Boxplots with Tukey Whiskers, unless otherwise stated.

## Figures and Tables

**Table 1:** Baseline characteristics.

	ESUS	AF (*N* = 25)		Angiopathic Stroke	*p*-Value
		Previously Diagnosed	Newly Diagnosed		
*N*	43	9	16	20	
Age (a) (IQR)	65.00 ^$$^ (52–75)	83.00 **^ßß^ (74–91)	77.50 (70–82.25)	68.50 ^§§^ (59.50–78.00)	**0.001**
Male	28 ^$^ (65%)	3 ^$^ (33%)	15 *^§^ (94%)	13 (65%)	**0.019**
Female	15 ^$^ (35%)	6 ^$^ (66%)	1 *^§^ (6%)	7 (35%)	
ESRS (IQR)	2.00 (1–3)	3.00 (2.5–3.5)	3.50 (2.25–4)	3.00 (2–4)	**0.016**
CHA_2_DS_2_ VASC (IQR)	4.00 ^$^ (3–5)	6.00 * (5–7)	6.00 (5–7)	5.00 (4–6.75)	**0.001**
Previous ischemic stroke	8 (19%)	1 (11%)	3 (19%)	8 (40%)	0.199
BMI (kg/m^2^) (IQR)	26.06 (23.15–28.76)	24.45 ^$ß^ (23.42–25.77)	26.23 ^§^ (24.69–29.91)	27.49 ^§^ (24.93–31.38)	0.108
NIHSS on admission (IQR)	2 (1–5)	4.00 (2–15.5)	4.00 (1.25–6.75)	3.00 (2–5)	**0.045**
Symptom to venous puncture time (days) (IQR)	7.00 (6–7)	7.00 (7–7)	7.00 (6–7)	7.00 (7–8)	0.127
Serum creatinine (µmol/L) (IQR)	78.00 (65–93)	75.00 (61–108)	89 (74–112)	81.50 (71.50–93)	0.215

The factors age, arterial hypertension, diabetes mellitus, previous myocardial infarction, other cardiovascular disease, peripheral arterial disease, nicotine consumption, previous stroke, and transient ischemic attack are subsumed in the ESRS. The factors age, sex, congestive heart failure, hypertension, history of Stroke/TIA/thromboembolism, other vascular disease, and diabetes mellitus are subsumed in the CHA_2_DS_2_VASC. */** indicates significant differences with the ESUS group. §/§§ indicates significant differences with the previously diagnosed AF group. $/$$ indicates significant differences with the newly diagnosed AF group. ß/ßß indicates significant differences with the angiopathic stroke group. *p*-values were calculated using the Mann–Whitney *U*-test, Kruskal–Wallis test or Chi-square test, as appropriate. *p* ≤ 0.05 was considered significant (bold).

**Table 2:** Comparison of Dimethylarginines and Carotid Intima–Media Thickness (CIMT) between ESUS and AF.

	ESUS	Newly Diagnosed AF		AF (Total)	
L-arginine (µmol/L) (median (IQR))	85.80 (69.70–106.10)	73.25 (58.40–88.80)	***p* = 0.03**	74.40 (57.24–93.52)	***p* = 0.031**
ADMA (µmol/L) (median (IQR))	0.48 (0.41–0.55)	0.51 (0.46–0.56)	*p* = 0.223	0.50 (0.46–0.56)	*p* = 0.26
SDMA (µmol/L) (median (IQR))	0.49 (0.41–0.61)	0.56 (0.52–0.77)	***p* = 0.026**	0.58 (0.52–0.80)	***p* = 0.004**
L-arginine/ADMA ratio (median (IQR))	176.51 (153.39–236.50)	148.75 (120.83–171.65)	***p* = 0.009**	148.64 (121.68–181.60)	***p* = 0.006**
L-arginine/SDMA ratio (median (IQR))	177.84 (130.85–222.12)	126.17 (84.99–162.44)	***p* = 0.004**	118.03 (80.16–175.79)	***p* = 0.002**
ADMA/SDMA ratio (median (IQR))	0.96 (0.77–1.17)	0.85 (0.68–0.95)	***p* = 0.046**	0.83 (0.67–0.95)	***p* = 0.013**
CIMT (mm) (median (IQR))	0.66 (0.57–0.75)	0.83 (0.69–0.91)	***p* = 0.001**	0.85 (0.76–0.92)	***p* < 0.001**

*p*-values were calculated using the Mann–Whitney *U*-test comparing ESUS and newly diagnosed AF or ESUS and AF (total). *p* ≤ 0.05 was regarded as significant (bold).

## Supplementary Materials

Supplementary materials can be found at http://www.mdpi.com/1422-0067/20/3/730/s1.

## Abbreviations

ADMA	asymmetric dimethylarginine
AF	atrial fibrillation
ASA	acetylsalicylic acid
AUC	area under the curve
BMI	body mass index
CI	confidence interval
CIMT	carotid intima–media thickness
DWI	diffusion-weighted imaging
ECG	electrocardiography
ESRS	Essen Stroke Risk Score
ESUS	embolic stroke of undetermined source
MRI	magnetic resonance imaging
NIHSS	National Institutes of Health Stroke Scale
NO	nitric oxide
NOAC	non-vitamin K oral anticoagulant
PFO	patent foramen ovale
ROC	receiver operating characteristic
SDMA	symmetric dimethylarginine
TEE	transesophageal echocardiography
