# Supplementary material for: Plasma Dimethylarginine Levels and Carotid Intima–Media Thickness are related to Atrial Fibrillation in Patients with Embolic Stroke
Source: Int J Mol Sci. 2019 Feb 9;20(3):730. doi: 10.3390/ijms20030730 (PMC6387438; doi:10.3390/ijms20030730)
Supplement: Supplementary file 1 [file ijms-20-00730-s001.pdf]

International Journal of *Molecular Sciences*

MDPI

Supplemental Material

Table 1. Correlation of markers of endothelial dysfunction with cardiovascular risk scores in the study collective.

		CHA_2DS_2 VASC	ESRS	L-arginine	ADMA	SDMA	L-arginine /ADMA Ratio	L-arginine /SDMA Ratio	ADMA/ SDMA Ratio	CIMT
CHA_2DS_2 VASC	r	1.000	**0.765****	−0.267*	**0.339****	**0.524****	**−0.480****	**−0.501****	**−0.334****	**0.499****
	p	.	0.000	0.012	0.001	0.000	0.000	0.000	0.001	0.000
	n	88	88	88	88	88	88	88	88	88
ESRS	r	**0.765****	1.000	−0.190	0.282**	**0.547****	**−0.380****	**−0.460****	**−0.388****	**0.497****
	P	0.000	.	0.077	0.008	0.000	0.000	0.000	0.000	0.000
	n	88	88	88	88	88	88	88	88	88

$p < 0.0028$ was regarded as significant after Bonferroni correction. Values were calculated using Spearman correlation.

Table 2. Correlation of markers of endothelial dysfunction with cardiovascular risk scores in patients with ESUS.

		CHA_2DS_2 VASC	ESRS	L-arginine	ADMA	SDMA	L-arginine /ADMA Ratio	L-arginine /SDMA Ratio	ADMA/ SDMA Ratio	CIMT
CHA_2DS_2 VASC	r	1.000	**0.745**	−0.285	0.437	**0.499**	**−0.572**	**−0.466**	−0.143	**0.455**
	p	.	0.000	0.064	0.003	0.001	0.000	0.002	0.359	0.002
	n	43	43	43	43	43	43	43	43	43
ESRS	r	**0.745**	1.000	−0.228	0.443	**0.615**	**−0.520**	**−0.531**	−0.279	0.390
	p	0.000	.	0.142	0.003	0.000	0.000	0.000	0.070	0.010
	n	43	43	43	43	43	43	43	43	43

$p < 0.0028$ was regarded as significant after Bonferroni correction. Values were calculated using Spearman correlation.

Table 3. Correlation of Arginine and Dimethylarginines with creatinine and eGFR.

		Arginine	ADMA	SDMA	L-arginine /ADMA Ratio	L-arginine /SDMA Ratio	ADMA/ SDMA Ratio	Creatinine	eGFR
creatinine	r	−0.275	0.132	**0.652**	**−0.350**	**−0.554**	**−0.621**	1.000	**−0.840**
	p	0.010	0.222	0.000	0.001	0.000	0.000	.	0.000
	n	88	88	88	88	88	88	88	88
eGFR	r	**0.375**	−0.257	**−0.756**	**0.527**	**0.689**	**0.628**	**−0.840**	1.000
	p	0.000	0.016	0.000	0.000	0.000	0.000	0.000	.
	n	88	88	88	88	88	88	88	88

$p < 0.0031$ was regarded as significant after Bonferroni correction. Values were calculated using Spearman correlation.
